# Trace element zinc metabolism and its relation to tumors

**DOI:** 10.3389/fendo.2024.1457943

**Published:** 2024-12-05

**Authors:** Guiping Yao, Zhiwei Wang, Rui Xie, Chenghao Zhanghuang, Bing Yan

**Affiliations:** ^1^ Department of Urology, Kunming Children’s Hospital, Kunming, Yunnan, China; ^2^ Department of Orthopedics, Kunming Children’s Hospital, Kunming, Yunnan, China; ^3^ Yunnan Province Clinical Research Center for Children’s Health and Disease, Kunming Children’s Solid Tumor Diagnosis and Treatment Center, Kunming, Yunnan, China; ^4^ Yunnan Key Laboratory of Children’s Major Disease Research, Yunnan Clinical Medical Center for Pediatric Diseases, Kunming Children’s Hospital, Kunming, Yunnan, China

**Keywords:** zinc, zinc metabolism, metabolic syndrome, cancer therapy, controversy

## Abstract

Zinc is an essential trace element in the human body, playing a crucial role in cellular metabolism.Dysregulation of zinc homeostasis can lead to abnormal cellular metabolism, contributing to diseases and closely related to tumor development. Adequate zinc intake can maintain zinc homeostasis in the body and support normal cellular metabolism. This review discusses the metabolic processes of zinc in the human body and its close relationship with tumorigenesis. It briefly describes zinc absorption, transport, storage, and release, as well as its important role in gene expression, signal transduction, oxidative stress, immune response, and apoptosis. It focuses on the abnormal cellular metabolism caused by excessive or insufficient zinc, the relationship between zinc homeostasis disruption and metabolic syndrome, and the mechanisms involved in tumor development. It analyzes how changes in the expression and activity of zinc transporters may lead to disrupted zinc homeostasis in tumor tissues. It points out that zinc deficiency is associated with various cancers, including prostate cancer, hepatocellular carcinoma, pancreatic cancer, lung cancer, ovarian cancer, esophageal squamous cell carcinoma, and breast cancer. The summary emphasizes that zinc metalloproteins could serve as potential targets for cancer therapy, and regulating the expression and activity of zinc transport proteins may offer new methods and strategies for clinical cancer treatment.

## 1 Introduction

Zinc is a vital trace element for the human body, playing a key role in protein composition within cells and participating in metabolic processes. Zinc is involved in the conformation and function of nuclear transcription factors, facilitating protein synthesis, and it also acts as a component of superoxide dismutase (SOD), providing strong antioxidant activity. Additionally, zinc plays a role in processes such as apoptosis and immune response (1). Dysregulation of zinc homeostasis can lead to disturbances in cellular metabolic functions and human diseases, with the relationship between zinc metabolism disorders and metabolic syndrome and tumors needing further investigation. However, there is currently no definitive conclusion regarding the correlation between zinc metabolism and metabolic syndrome, as existing studies show contradictory results, and the specific mechanisms supporting their relationship remain unclear. More research is needed to explore this issue, as numerous studies have proposed a link between zinc homeostasis disruption and cancer (2). This article briefly summarizes zinc absorption, transport, and its involvement in metabolic processes within the human body. It summarizes diseases related to abnormal zinc metabolism and focuses on the relationship between zinc homeostasis disruption, metabolic syndrome, and tumors. It discusses issues and controversies regarding zinc in cancer treatment, offering insights for cancer diagnosis and therapy.

## 2 Zinc absorption, transport, and its functions

The main source of zinc in the human body is from dietary intake, and it can also be transported from reserves in the liver, muscles, and other tissues to other parts of the body. In the stomach, zinc forms complexes with proteins in food under the action of gastric acid. It is mainly absorbed in the upper part of the small intestine through zinc transporters on the apical membrane of intestinal epithelial cells, particularly the ZIP4 transporter. After absorption, zinc is transported into the plasma through zinc ion channels and the ZnT1 transport protein located on the basolateral membrane of intestinal epithelial cells, and subsequently delivered to body tissues (3). Zinc participates in vital cellular activities (4). It is primarily found in the liver, pancreas, muscles, bones, and prostate, playing significant roles in human health.

**Figure 1:**
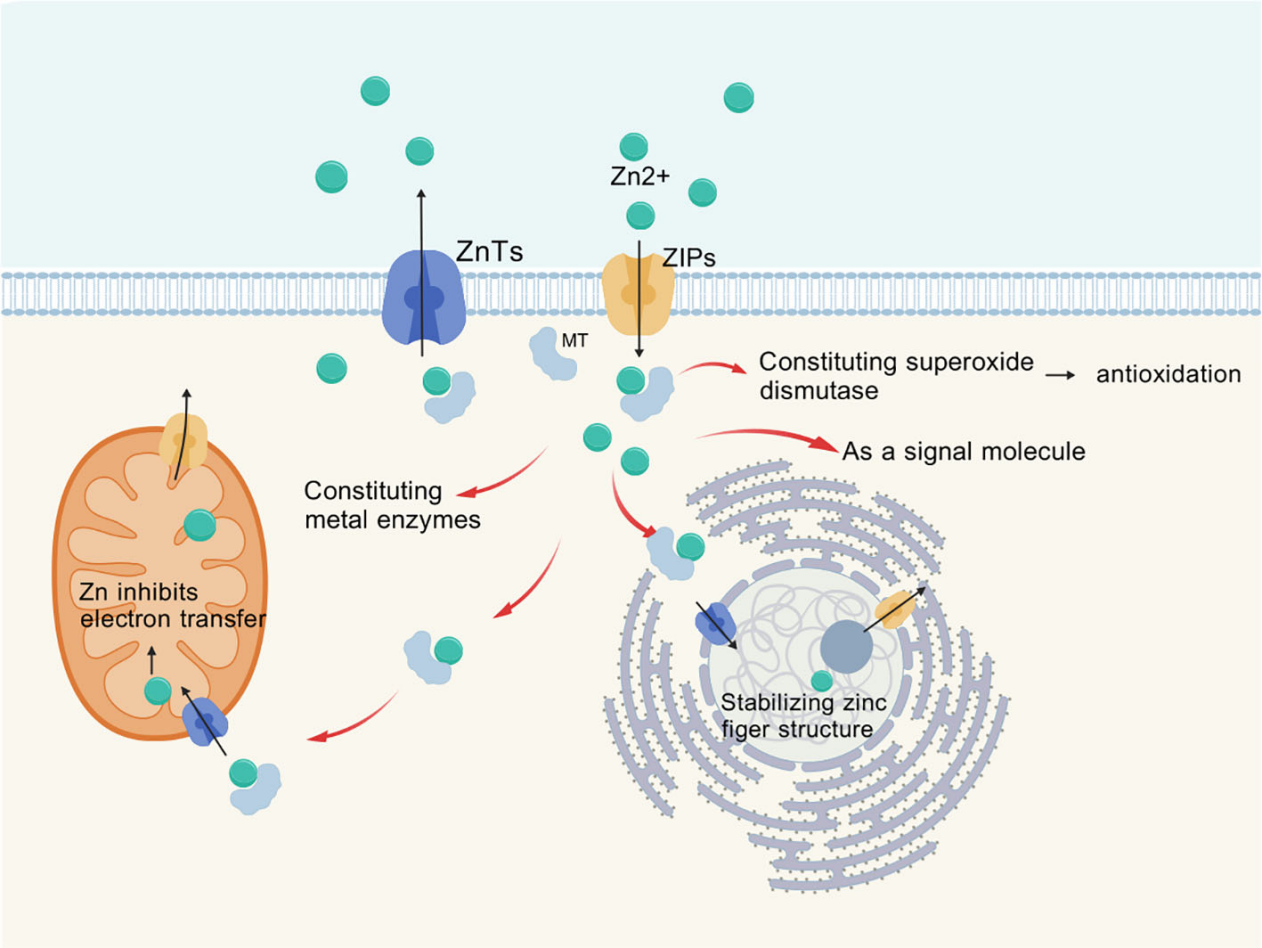
Transmembrane transport of Zinc via ZIPs, ZnTs.

Zinc is transported across membranes via transporters ZnT or ZIP (as shown in **Figure 1**), with ZIP family proteins facilitating the influx of zinc ions from the extracellular space or from intracellular vesicles and organelles into the cytoplasm (5). Currently, at least 14 types of ZIP proteins and 10 types of ZnT proteins have been identified in the human body, which exhibit tissue-specific differential expression (6). These zinc transporters exhibit structural homology as well as differences, and their varying tissue distribution and functions have been a popular research topic (7). Zinc binds to metallothioneins (MT) in the cytoplasm, which consist of four subtypes; MT-1 and MT-2 are present in all cells of the body, regulating intracellular levels and flux of zinc and copper while detoxifying heavy metals. MTs are also involved in nuclear transcription and play a role in immune function through their chelation of metals (8).

Zinc homeostasis within cells is maintained by the ZIP (zinc transporter) and ZnT (zinc transport protein) families, as well as metallothioneins (1, 9). These proteins are all regulated during their functions and interact with other metabolic and signaling pathways. Once zinc ions enter the cells, they are transported to various organelles, with a significant presence in mitochondrial metalloproteins. Many zinc metalloproteins are secreted or reside in organelles such as the endoplasmic reticulum, Golgi apparatus, and secretory vesicles (1). Zinc ion signaling regulates cellular proliferation, differentiation, ion transport, and secretory functions (10).

Zinc exists in two forms within human cells (11). One form of zinc is bound to proteins, serving as an essential component of many proteins in the human body (12). It can chelate with negatively charged parts of molecules, such as cysteine and histidine, providing structural bridges to maintain the three-dimensional conformation of polypeptides, known as zinc metalloproteins or zinc metallozymes. Currently, over 3,000 types of such metalloproteins have been identified (1). This includes oxidoreductases, transferases, hydrolases, lyases, isomerases, and ligases, which are involved in processes such as oxidative stress, apoptosis, and immune responses within cells (12). The other form is mobile zinc bound to non-protein ligands, the nature of which is still unknown (13). This type of zinc ion forms a loose association with non-protein ligands, known as free zinc ions, which serve as a pool for exchangeable zinc (11).

Zinc plays a crucial role in metabolic processes such as gene expression, signal transduction, oxidative stress, immune response, and apoptosis. It is involved in the conformation and function of nuclear transcription factors, such as zinc fingers and nuclear receptors, both of which are stabilized by four coordinated zinc ions. By stabilizing zinc finger structures, zinc executes important functions in cells, playing significant roles in DNA replication and repair, transcription and translation, cellular proliferation and maturation, as well as apoptosis regulation (14). Zinc, as a component of superoxide dismutase (SOD), exhibits strong antioxidant activity. SOD exists in three isoenzymatic forms in the body: copper-zinc superoxide dismutase, found in the cytoplasm; manganese superoxide dismutase (Mn SOD), located in the mitochondria; and extracellular superoxide dismutase (EC-SOD), which is present in extracellular spaces and fluids (15). These three forms of SOD work together to protect cells from the toxic effects of excessive reactive oxygen species. Zinc also acts as a cofactor in the formation of active thymosin (Zn FTS) released by thymocytes (9). Zn FTS regulates the differentiation of mature T cells in the thymus and the function of mature T cells in peripheral blood, having less impact on B cell development compared to T cells (16). It promotes the host defense functions of the immune system.

## 3 Disorders related to zinc homeostasis imbalance

Both excess and deficiency of zinc can lead to dysregulation of zinc homeostasis, and ultimately resulting in human diseases. Excessive zinc intake releases soluble zinc salts in acidic gastric fluid, which can directly irritate the gastrointestinal mucosa and lead to ulcer formation (17). This condition manifests as symptoms such as nausea, vomiting, decreased appetite, abdominal cramps, and headaches (18). Excessive zinc, once absorbed into the bloodstream, inhibits normal metabolic processes involving zinc (19). For example, high levels of zinc in the blood can suppress the antioxidant pathways in red blood cells, leading to oxidative damage to the cell membranes, which increases the risk of hemolysis, coagulopathy, and even triggers disseminated intravascular coagulation (DIC) (20). Systemic hypoxemia can then lead to liver dysfunction, pancreatitis, coagulation-related disorders, acute renal failure associated with tubular damage, and neurological abnormalities (19).

**Figure 2:**
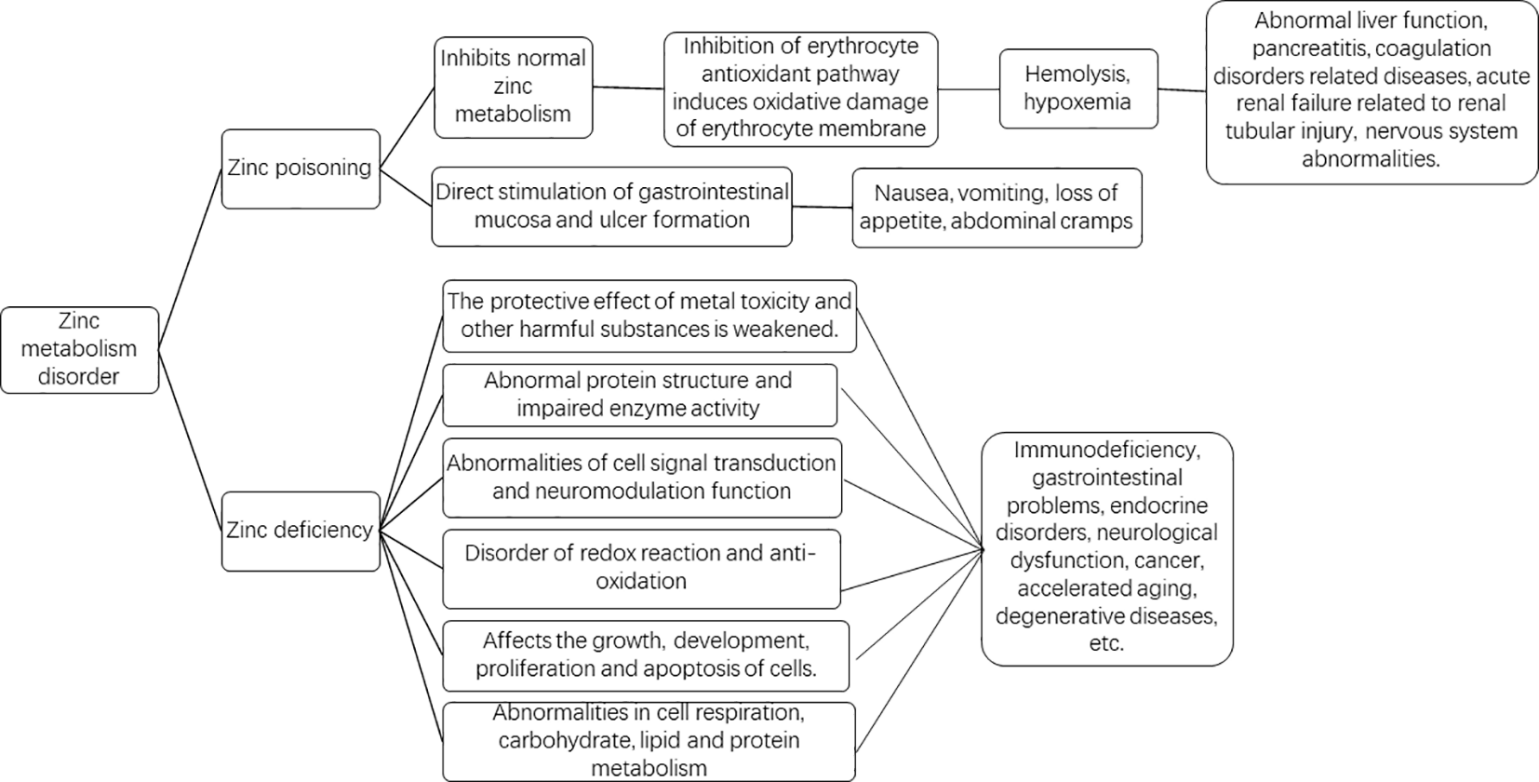
Graphic abstract of metabolic abnormalities caused by Zinc homeostasis disorder.

Mild micronutrient deficiencies in the human body can lead to chronic and subtle metabolic disturbances, resulting in DNA or mitochondrial damage, which may accelerate aging and contribute to cancer and degenerative diseases (21). Zinc deficiency is associated with diabetes, cirrhosis, inflammatory bowel disease, malabsorption syndrome, and sickle cell anemia (22–28). Zinc deficiency can lead to or exacerbate issues such as immune deficiencies, gastrointestinal problems, endocrine disorders, neurological dysfunction, cancer, aging, and degenerative diseases (19). Zinc deficiency affects all metabolic processes involving zinc (**Figure 2**), as evidenced by several aspects: first, certain intracellular transcription factors, hormone receptors, and many enzymes require zinc to maintain structural integrity. Zinc stabilizes the tertiary folding of smaller proteins, thereby contributing to the maintenance of their functional activity (29). Zinc also plays a structural role in ribosomes, cell membranes, and nucleic acids (30). Zinc deficiency can lead to protein structural abnormalities and impaired enzyme activity. Secondly, like calcium or nitric oxide (NO), zinc acts as an intracellular and intercellular messenger, activating intracellular signaling pathways and altering gene expression patterns. Free or exchangeable zinc (loosely bound zinc) can serve as a second messenger to control various functions, including gastric acid secretion, hormone release, and cardiac electrophysiology (31). Zinc, as a signaling molecule, functions similarly to other neurotransmitters and possesses neuromodulatory capabilities (32). In the immune system, zinc plays a role in intracellular, extracellular, and intercellular signaling. A classic sign of zinc deficiency in humans is impaired innate and cell-mediated immune functions, characterized by thymic atrophy, lymphopenia, reduced leukocyte function, and recurrent infections (33). Zinc deficiency leads to abnormalities in cellular signaling and neuromodulatory functions. Thirdly, redox reactions and antioxidant activities become dysregulated. Zinc can directly protect cell membranes from oxidative damage (34). Zinc can also indirectly reduce potential free radical formation and lipid peroxidation, as well as oxidative damage to proteins and DNA (34). Zinc deficiency is associated with increased oxidative stress factors and inflammatory biomarkers. Zinc supplementation can reduce biomarkers of oxidative stress, such as thiobarbituric acid reactive substances (TBARS) and malondialdehyde (MDA) levels (35). Most reactive oxygen species (ROS) are produced by NADPH oxidase (NOX), and zinc can scavenge ROS, exerting antioxidant effects. Zinc levels influence the activity and levels of copper-zinc superoxide dismutase (36). Zinc deficiency leads to abnormal thiol redox status in cell membranes, resulting in increased permeability and fragility of red blood cell membranes, as well as inactivation of calcium channel proteins in the membranes (30). The activities of antioxidant enzymes such as Cu/Zn superoxide dismutase, catalase, and peroxidase are affected, leading to weakened antioxidant defenses and accelerating the process of mitochondrial oxidative aging (37). Fourth, the processes of cell growth, development, proliferation, and apoptosis become dysregulated. Zinc deficiency affects the structure of key enzymes and transcription factors involved in DNA repair and replication, including DNA polymerases, DNA-dependent RNA polymerases, and reverse transcriptases (38). Abnormal formation of zinc finger proteins, such as transcription factors, transcriptional repressors, steroid receptors, thyroid receptors, vitamin D receptors, and retinoic acid receptors occurs (39). These enzyme and protein abnormalities contribute to disruptions in cellular growth, development, and proliferation. Zinc deficiency also promotes apoptosis, primarily in rapidly growing tissues such as intestinal crypt cells, the thymus, and other embryonic and fetal tissues (40). Fifth, the metabolism of carbohydrates, lipids, and proteins, as well as cellular respiration, becomes abnormal. Within mitochondria, zinc inhibits mitochondrial respiration, terminal oxidation, and ATP production by altering the function of mitochondrial enzymes and the cytochrome electron transport chain (41). Zinc-containing enzymes and proteins participate in the metabolism of nucleic acids, proteins, carbohydrates, and lipids by interacting with hormone receptors, transcription factors, and enzyme systems (19). When zinc is deficient, the functions of the aforementioned metabolic enzymes are hindered. Additionally, zinc deficiency affects the regulatory roles of zinc-dependent metalloproteins or their antioxidant membrane-stabilizing effects, leading to diminished protection against metal toxicity and other harmful substances (42).

Currently, there is no definitive conclusion regarding the relationship between zinc metabolism-related diseases and metabolic syndrome. Metabolic syndrome is a cluster of conditions associated with obesity, hypertension, hyperglycemia, hyperlipidemia, and hyperuricemia (43). Some animal studies have suggested that zinc nanoparticles improve obesity-induced cardiovascular diseases by reducing blood pressure, oxidative stress, cardiac iron accumulation, insulin resistance, and inflammatory markers (44). Althanoon Zeina et al. also suggested that zinc supplementation in patients with metabolic syndrome is associated with improvements in systolic blood pressure, body mass index, and metabolic parameters, recommending the correction of zinc deficiency in these patients (45). These studies suggest that there may be a connection between zinc and factors associated with metabolic syndrome. However, current research remains controversial regarding whether there is a link between zinc metabolism and metabolic syndrome (46). Research shows that adequate dietary zinc consumption is linked to a decrease in the risk of MetS (47–49). A study in China has also indicated that zinc levels in children are indeed associated with components of metabolic syndrome (50). A meta-analysis of observational studies by Ding J et al. indicated that dietary zinc intake is negatively correlated with metabolic syndrome (46). Wu Y et al. found that higher blood zinc concentrations are associated with adverse changes in metabolic risk factors related to metabolic syndrome, particularly concerning BMI and LDL-c, and this relationship exhibits gender differences, mainly affecting women (51). In Aydogdu A’s study, serum zinc levels were significantly elevated in children with metabolic syndrome (52). Due to limited evidence, more well-designed prospective cohort studies are needed to clarify the relationship between serum zinc levels and metabolic syndrome. The specific mechanisms linking zinc to metabolic syndrome remain unclear; a case-control study suggested that higher serum zinc levels might be related to the number of metabolic factors, independent of BMI and insulin resistance (48). Research on this issue is limited, necessitating further studies to clarify the role of zinc status in the mechanisms associated with metabolic syndrome and to determine the optimal range of blood zinc levels in the body (51). In contrast, a cross-sectional study conducted in Iran on the relationship between serum zinc levels and metabolic syndrome in children and adolescents suggested that there is no association between serum zinc levels and metabolic syndrome in children (53). A case-control study by Ennes Dourado Ferro F et al. also indicated that there is no relationship between zinc nutritional status and biochemical markers of metabolic syndrome (54). In summary, current research on the relationship between zinc metabolism and metabolic syndrome is contradictory, and the specific mechanisms in studies supporting their correlation remain unclear, necessitating further investigation into this issue.

## 4 Zinc metabolism abnormalities and tumor

Approximately two-thirds of tumors in the human body are related to addictive behaviors, diet, lack of exercise, excessive sun exposure, or infections. Zinc can protect cells from damage caused by inflammation and oxidative stress, leading to the hypothesis that zinc has anti-cancer properties (55). As mentioned earlier, zinc stabilizes the structure of proteins, DNA, RNA, and ribosomes within cells, and regulates gene expression through zinc finger transcription factors, thereby altering the expression of different components in the DNA damage response (DDR) (56). Zinc deficiency is involved in various aspects of cancer cell generation and growth. Changes in zinc ion concentration can directly and specifically affect the activity of YY1 (YY1 is an intrinsically disordered transcription factor, a protein regulator of gene expression that has been shown to be related to the progression of many cancers), leading to altered gene expression patterns and potentially resulting in tumor transformation or progression (57). Zinc deficiency (ZD) is present in various tumors and affects their occurrence and progression. For instance, zinc levels in the serum or plasma of breast cancer patients are significantly reduced (58–61). The serum zinc levels are significantly reduced in patients with acute leukemia (62). Similarly, serum zinc levels are also significantly lower in bladder cancer patients, as well as in those with esophageal squamous cell carcinoma (ESCC), malignant prostate cancer, and ovarian cancer (63). This decrease in serum zinc levels may be due to the increased uptake by tumor cells and enhanced enzyme activity, leading to a higher demand for zinc in cancerous tissues (64). Conversely, some studies have found that higher toenail zinc levels in men are associated with an increased risk of prostate cancer (65).

### 4.1 Zinc metabolic abnormalities and tumor-related mechanisms

Disruption of zinc homeostasis is associated with tumor development, and the role of zinc varies across different types of cancer (66). Zinc indirectly affects tumor cells by influencing gene expression and cell survival, and directly impacts tumor cells by regulating the activation, function, and/or survival of immune cells (67). Under physiological conditions, Th2 and Th1 cells collaboratively engage in anti-tumor immunity, with cytokines like IL-4, IL-5, and IL-6 promoting B lymphocyte antibody synthesis and contributing to cancer prevention, and zinc is essential for activating this series of responses (67). Zinc deficiency disrupts processes such as oxidative stress, DNA damage, DNA repair, cell cycle, apoptosis, metabolic changes, microRNA expression, and inflammatory factors, thereby promoting cancer development (36). Zinc deficiency reduces the number of T and B cells in the thymus and bone marrow, increasing the body’s susceptibility to infections and weakening its defenses, which results in a higher incidence of tumors. Another potential mechanism by which zinc inhibits tumor growth is closely related to its suppression of the activity of the nuclear transcription factor NF-κB (68, 69). NF-κB in its active form induces the expression of approximately 200 genes, which are related to angiogenesis, metastasis, and cell proliferation. Zinc influences gene expression at the nuclear level by stabilizing structures and regulating various transcription factors, including NF-κB (69). The NF-κB transcription factor can enhance inflammatory responses, particularly by boosting the production of pro-inflammatory cytokines by macrophages. Zinc ions negatively regulate NF-κB activity through proteins like A20 with zinc finger structures and by reversibly inhibiting phosphodiesterase (PDE), thus suppressing inflammatory responses and cancer development. The tumor-suppressing effect of zinc is also related to its antioxidant properties. Established cancer cells generate large amounts of reactive oxygen species (ROS), and the clearance of these ROS relies on the activity of antioxidant enzymes. However, zinc can protect healthy cells from the cytotoxic and genotoxic effects of hydrogen peroxide, but in tumor tissues, it can exacerbate the toxicity of H2O2, leading to oxidative stress dysregulation in cancer cells (70). The mechanism of zinc and cancer is shown in **Table 1**.

**Table 1:** Literature summary table on the relationship between zinc and cancer.

Relationship between zinc and cancer	Zinc proteins	Action mechanism	Reference
Carcinogenic effect of zinc	Zinc finger protein(ZNFs)	Overexpression of ZNF322A activates genes related to metastasis, tumor stemness and angiogenesis, thereby promoting the progression of lung cancer.	(71)
		Oncogenic zinc finger protein ZNF687 activates PI3K/Akt/mTOR signaling pathway to accelerate lung adenocarcinoma cell proliferation and tumor progression.	(72)
		Zinc finger protein CXXC5 promotes breast cancer by regulating TSC1/mTOR signaling pathway.	(73)
		GATA zinc finger protein p66β acts as a co-activator of Snail to promote breast cancer cell migration	(74)
		ZNF692 promotes proliferation, migration and invasion of osteosarcoma cells through TNK2-mediated MEK/ERK pathway activation.	(75)
		ZNF554 inhibits the progression of endometrial cancer by regulating RBM5 and inactivating WNT/β-Catenin signaling pathway.	(76)
		Zinc finger protein 263 promotes the malignant progression of non-small cell lung cancer by up-regulating interleukin 33 and inhibiting autophagy.	(77)
The anti-cancer effect of zinc	Metallothionein(MTs)	Activation of esterase D (ESD) promotes its interaction with MT2 A, reduces the protein level of MT2 A, up-regulates the concentration of free zinc ions, and inhibits the migration of A549 lung cancer cells *in vitro*.	(78)
		Metallothionein family proteins act as zinc ion regulators to synergistically enhance the anticancer effect of cannabidiol in human colorectal cancer cells.	(79)
	Zinc finger protein(ZNFs)	Zinc finger protein ZNF575 promotes the transcription of p53 to inhibit the growth of colorectal cancer.	(80)
		The protein expression of ZNF746 is significantly increased in colorectal cancer. ZNF746 plays an important role in the invasion and migration of colorectal cancer (CRC) cells.	(81)
		Zinc finger protein 671 plays a tumor suppressor role in colorectal cancer by inhibiting Notch signaling pathway.	(82)
	–	Zinc exerts its anti-tumor effect by acting on the central cytotoxic T cells of cellular immunity.	(83)
		Zinc deficiency promotes the proliferation, migration and invasion of esophageal squamous cell carcinoma EC109 cells.	(84)

Altered expression levels of zinc transporters are one of the reasons for zinc homeostasis disruption in tumor tissues (85). For example, current studies show that zinc levels are high in prostate tissue cells, and this elevated intracellular zinc level facilitates the production and secretion of citrate in prostatic fluid, as well as aids normal cells in exerting cytotoxic effects to eliminate harmful cells. In contrast, prostate tumor cells exhibit reduced zinc levels, which may be due to low expression of the ZIP1 transporter protein, leading to low intracellular zinc and consequently promoting tumor cell proliferation (86, 87). In ER-positive breast cancer, elevated expression levels of ZIP7 increase zinc levels in ER-positive breast cancer cells. ZIP7 is activated by serine phosphorylation and is involved in the pathways that promote the progression of ER-positive breast cancer (88).

### 4.2 Zinc and tumor therapeutic targets

The mechanisms by which zinc homeostasis dysregulation leads to tumors have given rise to different anticancer targets for various cancers. Zinc transport proteins may serve as potential targets for cancer therapy, as modulating their function or zinc levels could offer new strategies for cancer treatment (89). Zinc transport proteins serve as targets for cancer therapy. Messenger RNA analysis in pancreatic cancer cells shows overexpression of ZIP4, while other ZIP variants are downregulated. ZIP4 promotes cell proliferation and tumor progression, and interfering with the RNA involved in the generation of ZIP4 can inhibit tumor cell proliferation and invasion; however, more research is needed to further explore the related mechanisms (90). Excess zinc accumulates in breast tumor cells, and this excess zinc has a toxic effect on them. Breast tumor cells increase the expression levels of ZnT2, which transports the excess zinc into vesicles, thereby reducing its toxic effects. Inhibiting the activity of ZnT2 in breast tumor cells can release excess zinc from these vesicles, resulting in cytotoxic effects on malignant breast cancer cells (91). In esophageal squamous cell carcinoma (ESCC), ZIP6 promotes cancer cell proliferation, invasion, and metastasis by increasing intracellular zinc levels, thereby activating the PI3K/AKT and MAPK/Erk pathways. Targeting ZIP6 may represent a potential strategy for treating the aggressiveness of ESCC (92). In various tumor cells, the expression levels of zinc transporters differ (see **Table 2**), and regulating the expression or activity of these transporters could also serve as a strategy for cancer treatment. The increased expression of MT has been linked to the proliferation rate of tumor cells (93), indicating that MT could be a potential target for future cancer suppression research (93). Zinc finger proteins (ZNFs) regulate the expression of various target genes, influencing tumor occurrence, progression, and patient prognosis. ZNFs are also expected to serve as new biological markers or therapeutic targets for malignant tumors (94).

**Table 2:** Expression of zinc transporters in different types of cancer.

Type of cancer	Expression of zinc transporters	Potential tumor markers
Pancreatic cancer	ZIP4 increased	ZIP4
Prostate cancer	ZIP1 ZIP4 decreased.	MTs
ER-positive breast cancer	ZIP7 increased	ZIP7
non-small cell lung cancer	ZIP4 increased, ZnTs decreased	ZIP4
Hepatocarcinoma	ZIP14 ZIP2 ZIP9 decreased	ZIP14
stomach and colon cancer	ZIP10 increased	
ovarian cancer	ZIP4 increased	ZIP4
advanced kidney cancer	ZIP10 increased	ZIP10
cervical cancer	ZIP7 increased	ZIP7
Nasopharyngeal carcinoma	ZIP4 increased	ZIP4
bladder cancer	ZnT1 increased	ZnT1
oral squamous cell carcinoma	ZIP4 increased	

### 4.3 The controversy of zinc supplementation in tumor treatment

Zinc supplementation is beneficial for the treatment of many tumors. Studies have found that zinc supplementation can induce cytotoxicity in pancreatic cancer cells and reduce their invasiveness (95). Zinc oxide nanoparticles can also promote apoptosis in liver and ovarian cancer cells by inducing autophagy (96, 97). Metal chelators can form stable complexes with metals, reducing the consumption of metal ions. The antibiotic chloroquine is a metal chelator that can chelate zinc, increasing intracellular zinc levels in tumor cells and enhancing anti-cancer effects (98). Metal chelating compounds (such as disulfiram, chloroquine, and dithiocarbamate derivatives) serve as coordination complexes targeting metals like copper, zinc, and gold in the ubiquitin-proteasome pathway, potentially acting as anti-cancer drugs (99). However, zinc chelation may also produce side effects. Some studies suggest that ferroptosis is a cell death mechanism that can be targeted for cancer treatment, but zinc chelation may inhibit this mechanism (100). This presents a major controversy in the use of metal chelates for cancer treatment, and further research is needed to confirm the impact of these side effects. The use of metal chelators should consider the specific characteristics of the cancer being treated. The benefits and risks of targeted therapies involving zinc in different types of tumors still require further investigation.

The application of zinc supplementation in cancer treatment is becoming increasingly common, but there remains significant controversy regarding its therapeutic use and effects for some tumors. Current studies have many shortcomings, and the benefits and risks of zinc supplementation for cancer treatment require further investigation. A study examining whether a combination of antioxidant vitamins and minerals can reduce the risk of skin cancer (SC) randomly assigned 7,876 French women and 5,141 French men to receive either a daily antioxidant capsule (containing 20 mg of zinc) or a matched placebo. With a median follow-up time of 7.5 years, the results indicated that zinc-containing antioxidant supplements had differential effects on SC incidence, increasing the risk in women but not in men (101). Taking prostate cancer, which has been extensively studied, as an example, there is considerable controversy regarding the therapeutic and preventive roles of zinc in this context. Numerous experimental studies have confirmed that the application of zinc derivatives and supplements can inhibit the proliferation, migration, and invasion of prostate cancer cells. However, some studies suggest that the efficacy of zinc supplementation in any form appears to be limited (87), primarily because malignant tumor cells with ZIP1 deficiencies cannot uptake and accumulate zinc from increased plasma zinc concentrations. Additionally, zinc supplementation formulations, especially those containing cadmium and lead, may have potential contaminants, and the bioavailability of different zinc compounds (such as sulfates, gluconates, and less commonly used citrates) varies (102). Some research has suggested that direct intratumoral injection of zinc can inhibit the growth of prostate cancer cells in xenograft mice (103), but the practicality of this intratumoral administration method in humans remains to be debated. Another controversial aspect of zinc’s use in prostate cancer treatment is that zinc levels in metastatic and late-stage hormone-independent prostate cancer have not been established, and effective treatments for advanced malignant prostate tumors and metastases are lacking. A large prospective cohort study found that low-dose zinc supplementation (1 to 24 mg/d) after diagnosis was associated with a reduced risk of lethal prostate cancer in men with non-metastatic prostate cancer (104). Conversely, another 30-year follow-up study indicated that daily supplementation of more than 75 mg of zinc or supplementation for more than 15 years could significantly increase the risk of lethal and aggressive prostate cancer (102). Therefore, the potential risks and benefits of low-dose zinc supplementation after diagnosis, as well as the duration of supplementation, require further investigation regarding prostate cancer survival.

In summary, the trace element zinc is involved in the formation of intracellular proteins and plays a crucial role in cellular processes such as gene expression, signal transduction, oxidative stress, immune response, and apoptosis. Both excess and deficiency of zinc can lead to metabolic abnormalities in cells, resulting in disease; therefore, it is essential to ensure an appropriate zinc intake to maintain zinc homeostasis and normal cellular metabolism. Current research on the relationship between zinc metabolism and metabolic syndrome is contradictory, and the specific mechanisms supporting their correlation remain unclear, necessitating further studies to explore this issue. Cancer is a major disease impacting human health, and zinc homeostasis is closely related to cancer; zinc deficiency can disrupt cellular immune responses and oxidative stress, thereby promoting cancer development. Zinc supplementation has been shown to be beneficial for various cancers, including pancreatic, colorectal, liver, ovarian, and cervical cancers. The role of zinc in cancer varies by cancer type, allowing for the selection of different therapeutic targets based on specific mechanisms of action. The expression levels of zinc transport proteins vary across different cancer cells; thus, regulating the expression or activity of these transport proteins is also a therapeutic approach for cancer treatment. Many studies currently focus on zinc transport proteins as targets for cancer therapy, but further exploration is needed. Other proteins involved in zinc metabolism, such as metallothioneins and zinc finger structures, may also serve as potential research directions for future tumor suppressive targets, providing new approaches for clinical cancer treatment.
